# Whole-Genome Sequencing Identifies a Rice Grain Shape Mutant, *gs9–1*

**DOI:** 10.1186/s12284-019-0308-8

**Published:** 2019-07-18

**Authors:** Liangrong Jiang, Guotian Li, Mawsheng Chern, Rashmi Jain, Nhan T. Pham, Joel A. Martin, Wendy S. Schackwitz, Juan Zhao, Deling Ruan, Rongyu Huang, Jingsheng Zheng, Pamela C. Ronald

**Affiliations:** 1 Xiamen Plant Genetics Key Laboratory, School of Life Sciences, Xiamen University, Xiamen, 361102 People’s Republic of China; 2 Department of Plant Pathology and the Genome Center, University of California, Davis, CA 95616 USA; 3 Feedstocks Division, Joint BioEnergy Institute, Lawrence Berkeley National Laboratory, Berkeley, CA 94720 USA; 4 Biological Systems and Engineering Division, Lawrence Berkeley National Laboratory, 1 Cyclotron Road, Berkeley, CA 94720 USA; 5 State Key Laboratory of Agricultural Microbiology and College of Plant Science and Technology, Huazhong Agricultural University, Wuhan, 430070 Hubei China; 6 U.S. Department of Energy Joint Genome Institute, Walnut Creek, CA 94598 USA

**Keywords:** *Oryza sativa* L., Whole-genome sequencing, Grain shape, Fast-neutron-induced mutant population, Kitaake mutant database

## Abstract

**Background:**

Breeding for genes controlling key agronomic traits is an important goal of rice genetic improvement. To gain insight into genes controlling grain morphology, we screened M_3_ plants derived from 1,000 whole-genome sequenced (WGS) M_2_ Kitaake mutants to identify lines with altered grain size.

**Results:**

In this study, we isolated a mutant, named fast-neutron (FN) 60–4, which exhibits a significant reduction in grain size. We crossed FN60–4 with the parental line Kitaake and analyzed the resulting backcross population. Segregation analysis of 113 lines from the BC_2_F_2_ population revealed that the mutant phenotype is controlled by a single semi-dominant locus. Mutant FN60–4 is reduced 20% in plant height and 8.8% in 1000-grain weight compared with Kitaake. FN60–4 also exhibits an 8% reduction in cell number and a 9% reduction in cell length along the vertical axis of the glume. We carried out whole-genome sequencing of DNA pools extracted from segregants with long grains or short grains, and revealed that one gene, LOC_Os09g02650, cosegregated with the grain size phenotype in the BC_1_F_2_ and BC_2_F_2_ populations. This mutant allele was named *grain shape 9–1* (*gs9–1*). *gs9–1* carries a 3-bp deletion that affects two amino acids. This locus is a new allele of the *BC12/GDD1/MTD1* gene that encodes a kinesin-like protein involved in cell-cycle progression, cellulose microfibril deposition and gibberellic acid (GA) biosynthesis. The GA biosynthesis-related gene *KO2* is down-regulated in *gs9–1*. The dwarf phenotype of *gs9–1* can be rescued by adding exogenous GA_3_. In contrast to the phenotypes for the other alleles, the *gs9–1* is less severe, consistent with the nature of the mutation, which does not disrupt the open reading frame as observed for the other alleles.

**Conclusions:**

In this study, we isolated a mutant, which exhibits altered grain shape and identified the mutated gene, *gs9–1*. Our study reveals that *gs9–1* is a semi-dominant gene that carries a two-amino acid mutation. *gs9–1* is allelic to the *BC12/GDD1/MTD1* gene involved in GA biosynthesis. These results demonstrate the efficiency and convenience of cloning genes from the whole-genome sequenced Kitaake mutant population to advance investigations into genes controlling key agronomic traits in rice.

**Electronic supplementary material:**

The online version of this article (10.1186/s12284-019-0308-8) contains supplementary material, which is available to authorized users.

## Background

Grain weight, which encompasses length, width, length-to-width ratio, and thickness, is an important agronomic trait and a target for crop genetic improvement (Shi and Shen 1996; Tan et al. 2000). To date, more than 20 genes regulating rice grain shape have been isolated and characterized (Huang et al. 2013). These include *QTL for Seed Width on Chromosome 5* (*qSW5*), *Grain Length and Width on Chromosome 7* (*GLW7*), *Grain Width 7* (*GW7*), *Factor Slender Grain 3* (*SG3*)*, Grain Size and Number 1* (*GSN1*), *Grain Length 3.3* (*GL3.3*), *Grain Shape Gene on Chromosome 9* (*GS9*) and *gibberellin-deficient dwarf1* (*GDD1*) (Guo et al. 2018; Li et al. 2011; Shomura et al. 2008; Si et al. 2016; Wang et al. 2015, 2018; Xia et al. 2018; Zhao et al. 2018). To further investigate the genetic basis of grain weight, we screened the Kitaake mutant collection for mutants with altered grain shape and grain weight (Li et al. 2016).

Map-based cloning has been widely used since it was established in 1986. For this approach, researchers need to establish a large progeny population or near-isogenic lines, construct a genetic map, and carry out fine mapping to localize the gene (Kole and Gupta 2004; Sandal et al. 2005). Many genes have been successfully cloned using this approach. For example, the *Triticum aestivum* L *Reduced Height-D1* (*Rht-D1*) gene that controls plant height (Peng et al. 1999), the *Arabidopsis thaliana Fatty Acid Desaturation 3* (*FAD3*) gene encoding a omega-3 fatty acid desaturase (Arondel et al. 1992), the *Oryza longistaminata* Xanthomonas *21* (*Xa21*) gene for resistance against *Xanthomonas oryzae* pv. *oryzae* (Song et al. 1995), and the *Zea mays* teosinte glume architecture gene *Teosinte Glume Architecture1* were all isolated using map-based cloning (Wang et al. 2005). One drawback to the map-based cloning approach is that it requires extensive labor and time. With advances of next-generation sequencing (NGS) and comparative genomic analyses, gene cloning is now more efficient. Such an approach is particularly suitable for lethal mutants or mutants defective in seed setting which cannot be easily isolated using traditional map-based cloning approaches. For example, whole-genome sequencing (WGS) via NGS has been successfully applied to isolate genes from diverse species including the *Caenorhabditis elegans* neuronal cell fate decision gene *laterally* ***sy****mmetric-12* (Sarin et al. 2008), the *Drosophila melanogaster encore* (*enc*) gene that controls the egg morphology (Irvine et al. 2009), the *Schizosaccharomyces pombe* E2 ubiquitin ligase gene *ubiquitin conjugating enzyme 4* (*ubc4*) (Irvine et al. 2009), the *Bacillus subtilis* stringent response mediator gene (*relA*) (Srivatsan et al. 2008), the *A. thaliana* clock mutant early bird gene (*ebi-1*) (Ashelford et al. 2011), and the *O. sativa* the male sterility gene (*MER3*) (Chen et al. 2014).

With the advent of NGS, there has been renewed interest in isolating genes using mutant populations generated by chemical or irradiation mutagenesis. Over the years, several mutagens have been employed. For example, ethyl methane sulfonate (EMS) mutagenesis has been widely applied because of its simplicity and high efficiency in mutagenesis. Studies of EMS mutants show that there are estimated 1,499 SNPs in each rice mutant (Chen et al. 2014), and over 400 unique single nucleotide variants (SNVs) and 2 insertion/deletions (InDels) and copy number changes (CNVs) in each strain in *C. elegans* (Thompson et al. 2013). Obviously, EMS mutagenesis generates a high density of SNPs, which is important to reach mutation saturation but significantly increases the difficulty in cloning genes. Another approach to generate mutant populations is to use fast neutrons (FN) mutagenesis (Koornneeff et al. 1982; Li et al. 2001). This irradiation approach has been used to develop mutant populations in diverse plant species, including *A. thaliana* (Li et al. 2001), *Hordeum vulgare* (Zhang et al. 2006), *Citrus clementina* (Ríos et al. 2008), *Pisum sativum* (Domoney et al. 2013), *Glycine max* (Bolon et al. 2014) and *O. sativa* (Li et al. 2016, 2017). FN mutagenesis produces single base substitutions, deletions, insertions, inversions, translocations, and duplications (Bolon et al. 2014; Belfield et al. 2012; Li et al. 2016, 2017, 2001). In the Kitaake rice mutant population, an average of 59 mutations and 31 genes are affected in each rice line (Li et al. 2016). These reports suggest that the number of mutations in the Kitaake FN mutant population is smaller than that of the EMS lines (Chen et al. 2014; Thompson et al. 2013), making it more efficient to construct useful genetic populations to isolate the corresponding mutant allele.

In the present study, we described the characterization of FN60–4, a mutant altered in grain shape, which was identified upon visual inspection of M_3_ plants derived from 1000 WGS M_2_ Kitaake mutants (Li et al. 2016, 2017). We established three segregating populations by crossing mutant FN60–4 with Kitaake to create BC_1_F_2_, BC_2_F_2_ and BC_3_F_2_ populations. We analyzed grain shape, grain weight and plant height, in these populations, established wild-type and mutant gene pools and carried out WGS to identify a new allele of *GDD1* controlling grain shape, which we named *gs9–1*.

## Results

### Identification and Genetic Characterization of the *gs9–1* Allele

**Fig. 1:**
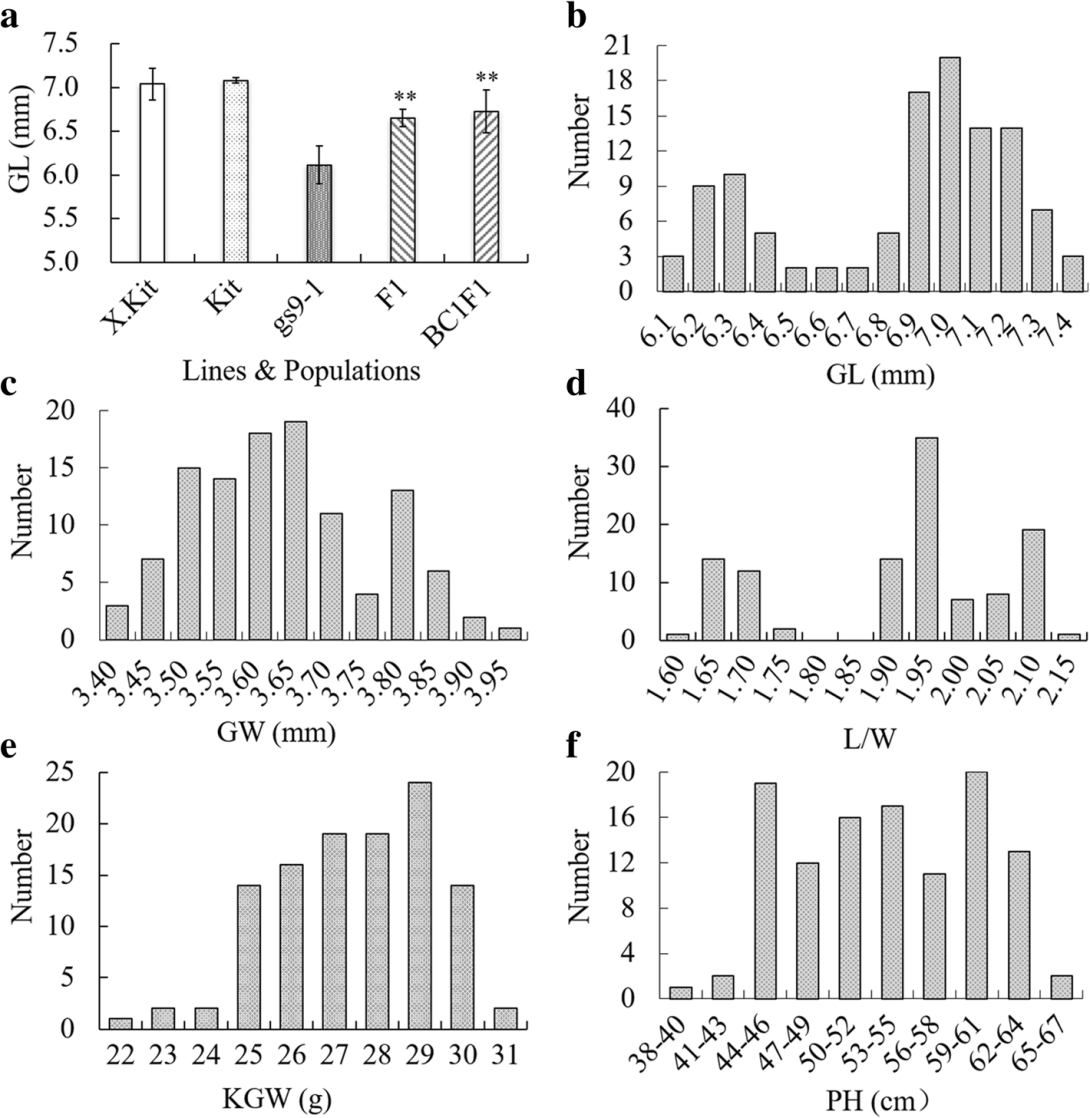
Grain length, grain width, grain length-to-width ratio, 1000-grain weight and plant height of the parental lines, mutant line, F_1_ and the BC_2_F_2_ population. GL, grain length; GW, grain width; L/W, grain length-to-width ratio; KGW, 1000-grain weight, PH, plant height. **a** The mean value of grain length of X. Kitaake (X.Kit), Kitaake (Kit), *gs9–1*, F_1_ (from the crossing between *gs9–1* and Kit) and BC_1_F_1_ (from the crossing between F_1_ and Kit). **b**-**f** Distribution of GL, GW, L/W, KGW and PH in the BC_2_F_2_ population. ** indicate the significant difference (*P* < 0.01) between the samples indicated using the unpaired Student’s *t*-test

We screened individual M_3_ plant derived from 1,000 independent M_2_ mutant lines for alterations in grain shape. From this screen, the *gs9–1* mutant exhibited the most significant reduction in grain length (Fig. 1). We then backcrossed the *gs9–1* mutant to its Kitaake parent, generating a segregating F_2_ population. Our measurements of grain shape revealed that the average grain length (GL) of plants X. Kitaake, Kitaake, *gs9–1*, F_1_ and BC_1_F_1_ is 7.04 mm, 7.08 mm, 6.12 mm, 6.65 mm and 6.73 mm, respectively. Grains from *gs9–1* show approximately 13.6% reduced length compared with Kitaake. F_1_ plants exhibited a medium grain length compared with the two parental lines (Fig. 1a; Additional file 1: Table S1).

We performed grain length (GL), grain width (GW), grain length-to-width ratio (L/W), 1000-grain weight (KGW) and plant height (PH) phenotypic analysis of a total of 113 lines from the BC_2_F_2_ population. The significance test of normal distribution based on IMB SPSS Statistics 19 show that GL (Fig. 1b), GW (Fig. 1c), and L/W (Fig. 1d) except KGW (Fig. 1e) and PH (Fig. 1f) are not normally distributed. GL displays a near double-peak distribution (Fig. 1b), and the GW (Fig. 1c) and L/W (Fig. 1d) both exhibit a three-peak distribution. We divided the BC_2_F_2_ population into three groups based on grain shape (L/W): wild-type group (WT group), L/W > 2.00; heterozygous type group (H group), 1.85 < L/W ≤ 2.00; and mutant group (M group), L/W ≤ 1.75. There are 28 lines in group WT, 56 lines in group H, and 29 lines in group M (Table 1). The chi square test indicates that the grain shape of this population fits the semi-dominant gene segregation ratio of 1:2:1 (χ_c_^2^ = 0.06 < χ_0.05,2_^2^, χ_0.05,2_^2^ = 5.99). There are highly significant differences in GL between the three groups, as well as in GW and L/W (Table 1). There is no significant difference in KGW between groups WT and H, while there are highly significant differences between group M and the other two groups, as well as in PH (Table 1). The average GL, GW, L/W, KGW and PH of the WT group are 7.27 mm, 3.52 mm, 2.06, 28.3 g and 55.5 cm, respectively, and those of the M group are 6.33 mm, 3.82 mm, 1.66, 25.8 g and 49.3 cm, respectively (Table 1). Compared with the WT group, the M group carried a 12.9% decrease in GL, 19.4% in L/W, 8.8% in KGW and 11.2% in PH, and an 8.5% increase in GW.

**Table 1:** Grain shape and plant height of three groups of the BC_2_F_2_ population

Group^a^	GL (mm)	GW (mm)	L/W	KGW (g)	PH (cm)	Plant Number
WT	7.27 ± 0.02^A^	3.52 ± 0.01^C^	2.06 ± 0.01^A^	28.3 ± 0.3^A^	55.5 ± 1.3^A^	28
H	7.01 ± 0.02^B^	3.64 ± 0.01^B^	1.92 ± 0.01^B^	27.9 ± 0.2^A^	55.0 ± 0.9^A^	56
M	6.33 ± 0.02^C^	3.82 ± 0.01^A^	1.66 ± 0.01^C^	25.8 ± 0.2^B^	49.3 ± 0.8^B^	29

^a^WT, H and M indicate wild-type, heterozygous and homozygous *gs9–1* genotypes, respectively. *GL* Grain length, *GW* Grain width, *L/W* Grain length-to-width ratio, *KGW* 1000-grain weight, *PH* Plant height. The same capital after the mean value between two groups indicates no significant difference and the different capitals between two groups indicate highly significant differences using the ANOVA analysis

**Fig. 2:**
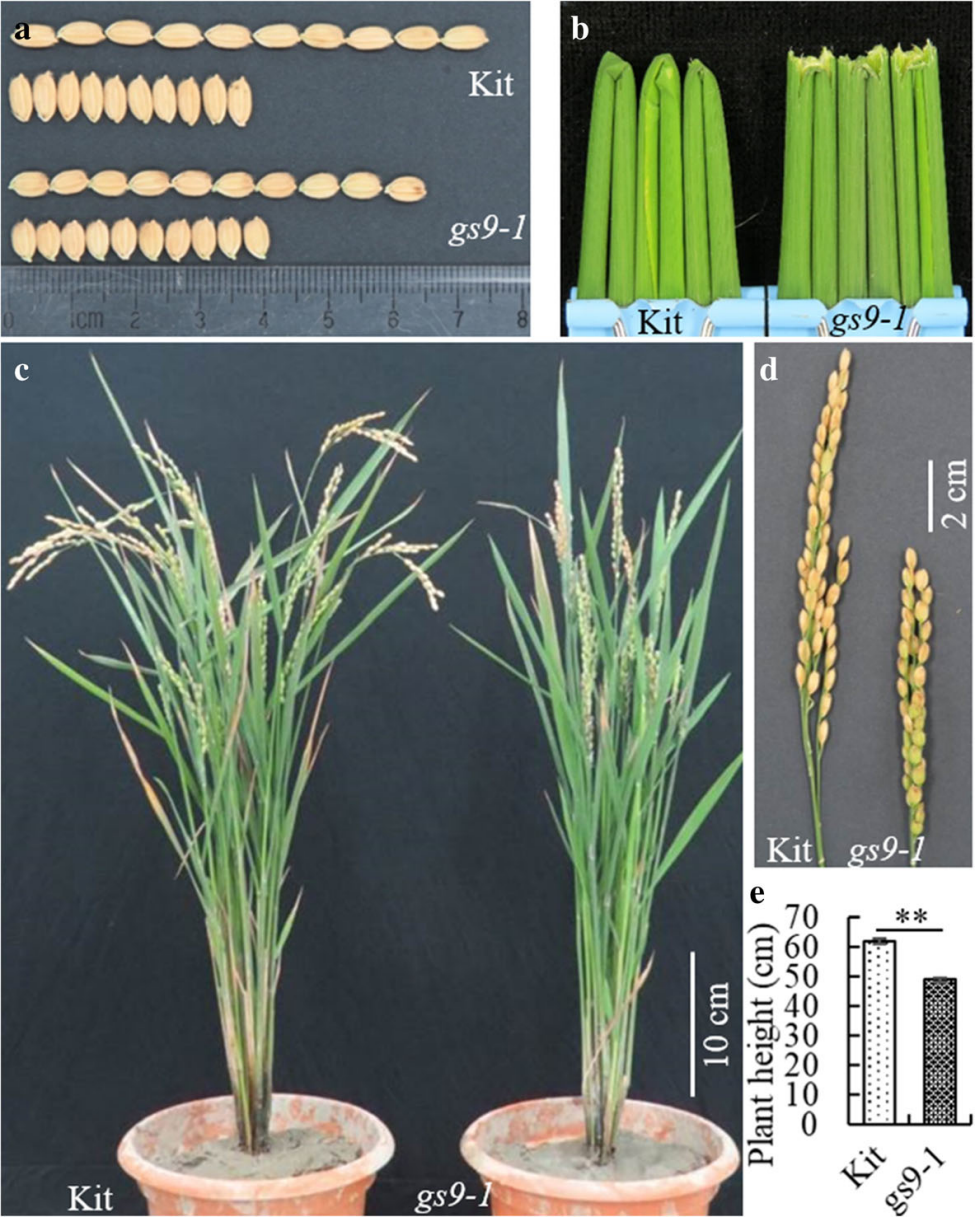
Grain, panicle and plant morphology of Kitaake and *gs9–1* plants. **a** Grains of *gs9–1* are shorter than those of Kitaake while grain width is slightly increased. **b** The culm brittle phenotype of *gs9–1*. **c** Plant stature of *gs9–1* is shorter than that of Kitaake. **d** Panicles of *gs9–1* are shorter than those of Kitaake. **e** Plant height. ** indicate the significant difference (*P* < 0.01) using the unpaired Student’s *t*-test

These findings suggest that mutant FN60–4 carries a single mutated gene/locus, named *grain shape 9–1* (*gs9–1*), which controls grain shape in a semi-dominant manner. This mutation leads to an obvious GL, KGW, panicle length and PH reduction, a GW increase and a brittle culm phenotype (Fig. 2).

To further validate these results, we analyzed GL, L/W, KGW, PH and GW in a BC_3_F_2_ population. Similar to the results of the BC_2_F_2_ population, we found that the M group carried an about 13.3% decrease in GL, 24.4% in L/W, 13.2% in KGW and 19.7% in PH, and a 7.3% increase in GW compared with the WT group (Additional file 1: Figure S1 and Table S2).

### Electron Microscopy Reveals the Changes in Cell Size and Number of Epidermal Cells of Grain Glume in the *gs9–1* Mutant

**Fig. 3:**
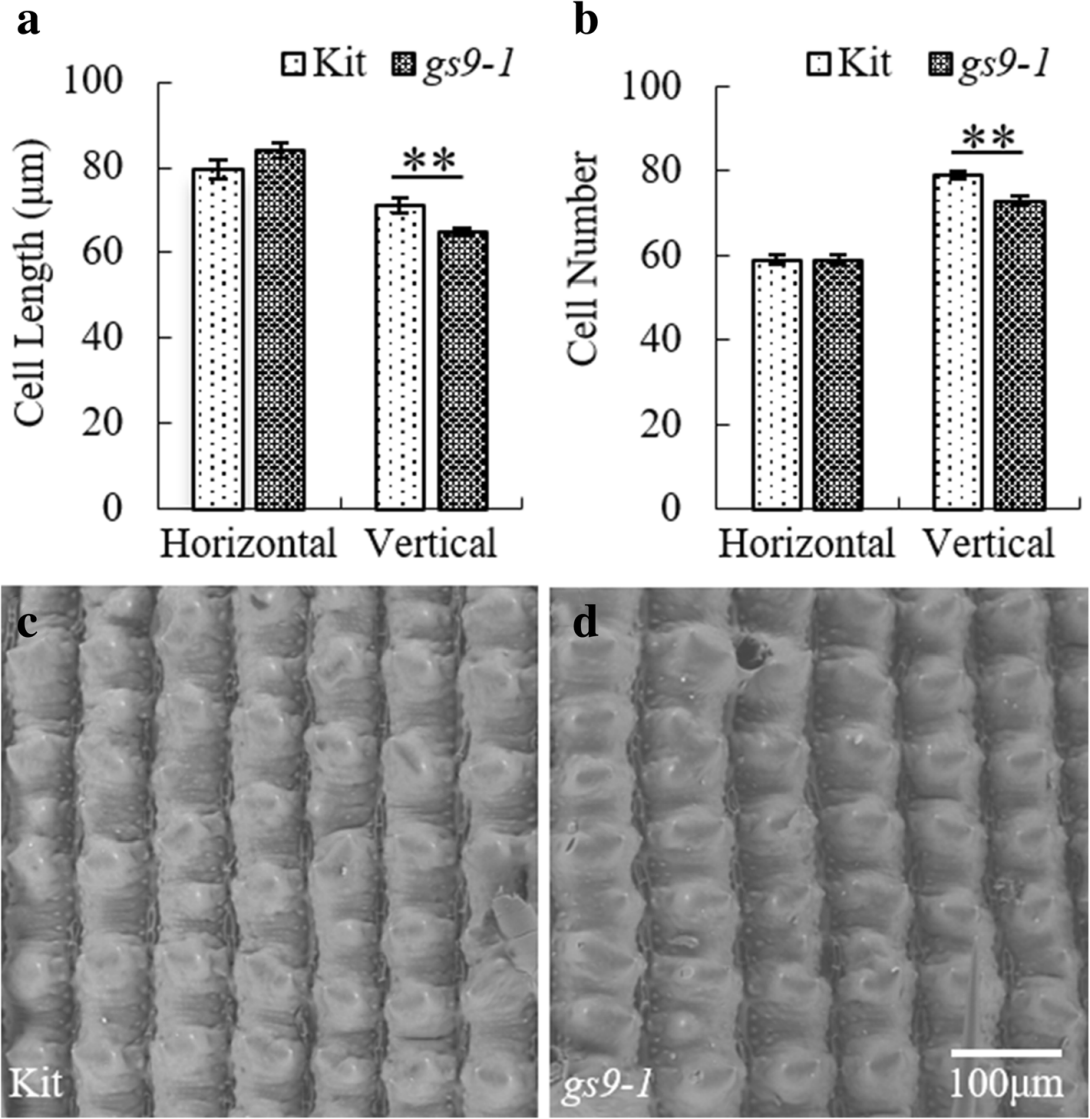
Analysis of the epidermal cell size and number of the *gs9–1* grain glume. **a** Cell length of grain on the horizontal axis and vertical axis. **b** Epidermal cell numbers of grain glume on the horizontal axis and vertical axis. **c** and **d** Epidermal cells of the Kitaake and *gs9–1* grain glume under the scanning electron microscope (× 300). ^**^ indicates an extremely significant difference using the unpaired Student’s *t-*test

To examine possible morphological changes at the cellular level, we assessed the cell morphology of *gs9–1* and Kitaake grains using scanning electron microscopy (SEM). Analysis of epidermal cells of grain glume showed that the mean cell length of grain glume on the horizontal axis (cell width) of Kitaake and *gs9–1* is 79.5 ± 2.1 μm and 84.0 ± 1.8 μm, respectively. The independent-samples T test shows no statistical difference between Kitaake and *gs9–1* basing on Statistical Product and Service Solutions (SPSS software) (*P* = 0.123) (Fig. 3a, c, d; Table 2). The mean cell length of Kitaake and *gs9–1* on the vertical axis (cell length) is 71.2 ± 1.7 μm and 64.8 ± 1.0 μm, respectively, with a statistically significant difference (*P* = 0.0037) (Fig. 3a, c, d; Table 2). The mean cell numbers of Kitaake and *gs9–1* on the horizontal axis are both 59 ± 1 (Fig. 3b; Table 2), and their mean cell numbers on the vertical axis are 79 ± 1 and 73 ± 1, respectively, representing a highly significant difference (*P* < 0.0001) (Fig. 3b; Table 2). These findings suggest that the cell number on the vertical axis of mutant *gs9–1* is significantly reduced, and cell length becomes shorter, whereas cell width of *gs9–1* increases slightly.

**Table 2:** Epidermal cell size and number of grain glume in Kitaake and *gs9–1* on the horizontal axis and vertical axis

Lines	Horizontal axis		Vertical axis	
	Cell width (μm)	Cell number	Cell length (μm)	Cell number
Kitaake	79.5 ± 2.1	59 ± 1	71.2 ± 1.7	79 ± 1
*gs9–1*	84.0 ± 1.8	59 ± 1	64.8 ± 1.0^a^	73 ± 1^a^

^a^The unpaired Student’s *t-*test shows that there are extremely significant difference between Kitaake and *gs9–1* using SPSS software

### Whole-Genome Sequencing Revealed All Mutations in Mutant FN60–4

We collected young leaves from two pools of the M_4_ population that were grouped based on short or long grain shape. Each of the pools, the long grain pool, named FN60–4H, and the short grain shape pool, named FN60–4 M, comprised 20 lines. Genomic DNA was pooled separately for FN60–4H and FN60–4 M. The pooled FN60–4H and FN60–4 M genomic DNA samples were sequenced on the Illumina HiSeq 2000 platform. FN60–4H had 153,804,078 aligned reads, providing a 40.71-fold coverage, and FN60–4 M had 158,085,074 aligned reads, giving a 41.93-fold coverage (Additional file 1: Figure S2; Table 3).

**Table 3:** Genome sequencing summary for gene pools used in this study

DNA pools	Raw sequencing data	Aligned sequencing data		Accession number
	Number of reads^a^	Number of reads	Fold coverage^b^	
FN60–4H	174,105,280	153,804,078	40.71	SRA186155
FN60–4 M	163,946,826	158,085,074	41.93	SRA237718

^a^DNA samples of all rice lines were sequenced using Illumina HiSeq 2000 platform sequencers to generate 100 bp paired-end reads

^b^The reference Nipponbare genome size of 374,471,240 bp was used to calculate the average sequencing depth

The whole-genome sequence alignment revealed 30 mutations in FN60–4H and 27 mutations in FN60–4 M. The 30 mutations in FN60–4H contain the 27 mutations FN60–4 M (Additional file 1: Table S3), composed of 13 single base substitutions (SBSs), 10 insertion-deletions (InDels) and 7 deletions (DELs, refers to the deletion of more than 10 base pairs). Among these mutations, six of the 16 homozygous mutations in FN60–4 M affect 19 genes, including five SNP or InDel mutations affecting LOC_Os01g44250 on chromosome 1, LOC_Os02g05150 on chromosome 2, LOC_Os04g22720 and LOC_Os04g53720 on chromosome 4, LOC_Os09g02650 on chromosome 9, in addition to a large deletion on chromosome 9 affecting 14 genes (Additional file 1: Table S3).

### *gs9–1* Carries a 3-bp Deletion in Gene LOC_Os09g02650 Affecting Two Amino Acids

**Fig. 4:**
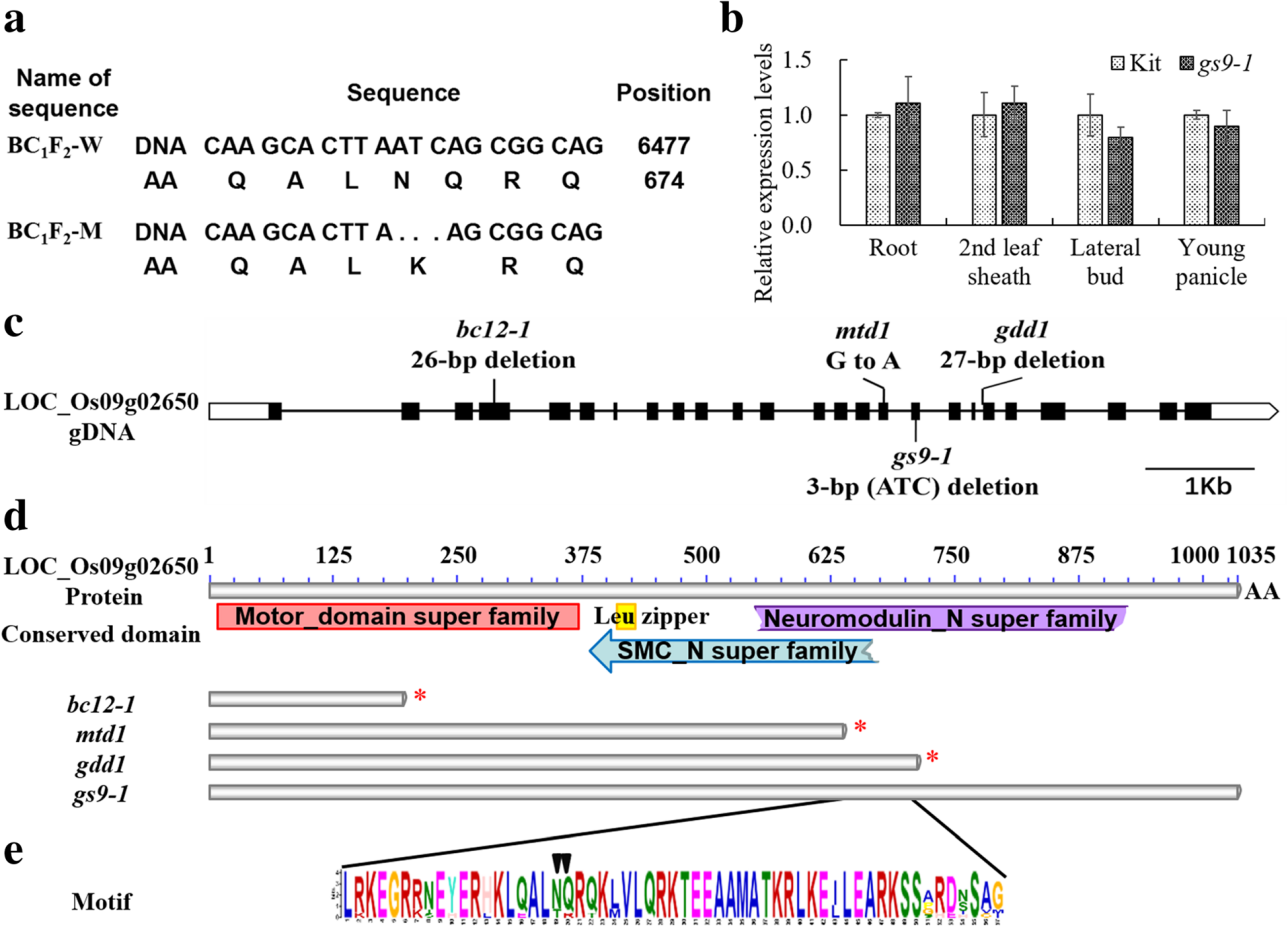
Gene and protein structures, and the relative expression of *GS9–1*. **a** The genomic and amino acid changes of line *gs9–1.* BC_1_F_2_-W is the wild-type pool, and BC_1_F_2_-M is the *gs9–1* mutant pool that carries a 3-base (ATC) deletion. The amino acid (AA) sequence alignment shows that amino acid lysine (K) in Kitaake is substituted by amino acids asparagine (N) and glutamine (Q) in *gs9–1*. **b** qRT-PCR assays of LOC_Os09g02650 in Kitaake and *gs9–1* in roots and the 2nd leaf sheath at seeding stage, the lateral bud at tillering stage and the young panicle at the fifth stage of panicle differentiation. The unpaired Student’s *t*-test showed that there is no different expression level between Kitaake and *gs9–1*. **c** Gene structure of LOC_Os09g02650 and the mutated sites of different alleles. **d** Conserved protein domains in LOC_Os09g02650 and protein structures of different mutant alleles. Motor domain, kinesin motor domain; SMC_N, the N terminal domain of the structural maintenance of chromosomes (SMC) proteins; Leu zipper, Leucine residues conserved in the bZIP protein; Neuromodulin-N, Gap junction protein N-terminal region. Information on putative conserved domains is retrieved from NCBI. ‘AA’ indicates amino acids. The asterisk indicates a premature translation termination. **e** The two mutated amino acids are indicated by black triangles in *gs9–1*. The height of a letter indicates this amino acid’s relative frequency at the given position (x-axis) predicated using the MEME program

A total of 19 genes in the previous 6 homozygous mutations were identified in the WT gene pool (BC_1_F_2_-W) and mutant gene pool (BC_1_F_2_-M) constructed from the BC_1_F_2_ population. Sequencing analysis revealed that LOC_Os09g02650 is unique to the BC_1_F_2_-M gene pool because it harbors a 3-bp deletion (ATC) in BC_1_F_2_-M but not in BC_1_F_2_-W (Fig. 4), indicating that this mutation possibly caused the mutated phenotype. In contrast, mutations in LOC_Os01g44250, LOC_Os02g05150, LOC_Os04g22720 or LOC_Os04g53720 do not cosegregate with BC_1_F_2_-M (Additional file 1: Table S4). We also designed 13 pairs of primers to amplify parts or total sequences of the 14 genes in the large deleted region at site 28,2372 bp of chromosome 9 (Additional file 1: Table S4). We examined 123 homozygous *gs9–1* progenies derived from a BC_2_F_3_ population, and found one line (V419, 19–5) that carried the same genotype as its parent Kitaake. This result excludes the possible involvement of this large deletion as being the cause of the *gs9–1* phenotype (Additional file 1: Figure S3; Additional file 2: Table S6). These results suggested that the gene responsible for the change of grain shape for *gs9–1* is LOC_Os09g02650. The 3-bp deletion in the 17th exon of *gs9–1* led to an amino acid change (N671K) and the deletion of the 672th amino acid residue Q (Fig. 4a, c). Three alleles of LOC_Os09g02650 have been previously isolated. These include *GDD1*, *Brittle Culm 12* (*BC12*), and *Multi-tillering Dwarf 1* (*MTD1*) (Li et al. 2011; Yu et al. 2016; Zhang et al. 2010). That dwarf phenotype observed for mutants *gdd1/mtd1* is due to the defects in GA biosynthesis (Li et al. 2011; Yu et al. 2016).

### qRT-PCR Assays of Gene LOC_Os09g02650

To test whether the mutation affects the expression of LOC_Os09g02650 in line *gs9–1*, we analyzed the relative expression level of gene LOC_Os09g02650 in roots, the 2nd leaf sheath at the seedling stage, the lateral bud at the tillering stage and a young panicle at the fifth stage of panicle differentiation using qRT-PCR. The results showed that there is no significant difference between WT and *gs9–1* in these tissues (Fig. 4b), excluding the possibility that the mutant traits are caused by the altered expression of gene LOC_Os09g02650.

### The Motif Analysis of LOC_Os09g02650 in Line *gs9–1*

To analyze whether potential paralogs of LOC_Os09g02650 in rice might affect the phenotype of *gs9–1*, we searched the rice genome using BLASTP with the full-length protein sequence of LOC_Os09g02650. We found no paralog of gene LOC_Os09g02650 (Additional file 2: Table S7). We searched for the conserved protein domain in gene LOC_Os09g02650 leading to the identification of four conserved domains: kinesin motor_domain, Leucine zipper, Neuromodulin_N and SMC_N domains (Fig. 4d). The mutation site of *gs9–1* is in the Neuromodulin_N domain. We analyzed putative 9 homologs of LOC_Os09g02650 from *A. thaliana*, *Sorghum bicolor*, *Zea mays*, *Panicum hallii*, *Setaria italica*, *Brachypodium distachyon* and *Triticum urartu* using MEME (Multiple Em for Motif Elicitation). We found that this gene is conserved and that the mutation site of line *gs9–1* is within a conserved protein motif (Neuromodulin_N) (Fig. 4e; Additional file 1: Figure S4), indicating that the mutated amino acids might be important for the proper function of gene LOC_Os09g02650.

### The *gs9–1* Mutant is Defective in GA Biosynthesis

**Fig. 5:**
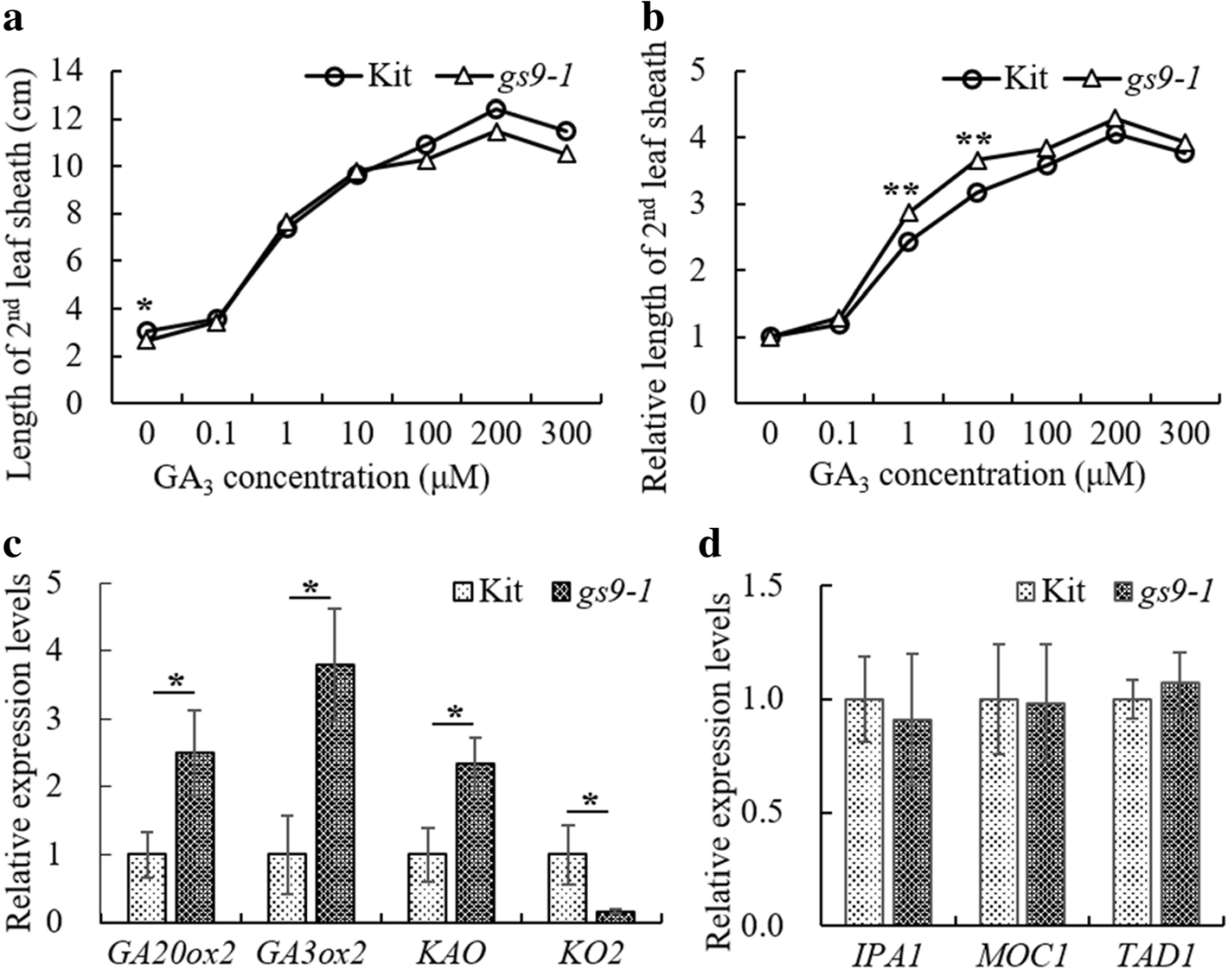
Response of *gs9–1* to GA_3_ and qRT-PCR assays of *gs9–1*. Asterisks indicate significant differences using the unpaired Student’s *t*-test (*P < 0.05; ** *P* ≤ 0.01). **a** GA_3_ response assays. Elongation of the second leaf sheath in *gs9–1* and Kitaake in response to GA_3_. **b** Relative length change of the 2nd leaf sheath, which was calculate by dividing the length of the 2nd leaf sheath stimulated with GA_3_ by that without GA_3_. **c** qRT-PCR assays of four genes involved in GA biosynthesis in the 2nd leaf sheath of *gs9–1* and Kitaake. The expression level of each gene from Kitaake was set to 1. The actin gene was used as the internal control. Data are means ± SD (*n* = 3). **d** qRT-PCR assays of three genes involved in rice tillering in lateral buds of *gs9–1* and Kitaake

Previous studies indicate that the dwarf phenotype of mutants *gdd1/mtd1* is due to the defects in GA biosynthesis (Li et al. 2011; Yu et al. 2016). To test if the mutant traits of the *gs9–1* mutant are involved in GA biosynthesis, we analyzed the response of the 2nd leaf sheath to exogenous GA_3_ at different concentrations. The 2nd leaf sheath length of line *gs9–1* is shorter compared to that of Kitaake without addition of GA_3_ but the slow growth of the 2nd leaf sheath was rescued when supplemented with 0.1–300 μM GA_3_ in the media (Fig. 5a). In comparing the relative growth, we observed that the *gs9–1* mutant responded to GA_3_ more robustly than Kitaake at concentrations of 1 and 10 μM (Fig. 5b). The finding indicates that exogenous GA_3_ can rescue the dwarf phenotype of line *gs9–1* and that line *gs9–1* might be defective in GA biosynthesis.

### qRT-PCR Assays of Genes Involved in GA Biosynthesis and Tillering

We further analyzed the expression levels of four representative genes involved in GA biosynthesis, including *KO2, GA20ox2/SD1*, *GA3ox2/D18*, and *KAO* in the 2nd leaf sheath of Kitaake and *gs9–1* at the 8-day stage. The expression levels of *GA20ox2/SD1*, *GA3ox2/D18*, and *KAO* are all significantly higher in *gs9–1* compared to that of Kitaake, while that of *KO2* is significantly down-regulated in *gs9–1* (Fig. 5c). This result suggests that the phenotype of *gs9–1* is possibly associated with defects in GA biosynthesis resulting from reduced expression of *KO2*.

We analyzed the expression levels of *IPA1*, *MOC1* and *TAD1,* which are involved in rice tillering in mutant *mtd1* (Yu et al. 2016). Their relative expression levels showed no difference between Kitaake and *gs9–1* (Fig. 5d), which is consistent with the fact that line *gs9–1* shows normal tillering.

## Discussion

In this study, we used phenotypic, genetic, physiological, and molecular evidence to demonstrate that *gs9–1* is a new allele of *BC12/GDD1/MTD1*. *BC12*/*GDD1/MTD1* encodes a dual-targeting kinesin protein (Li et al. 2011; Zhang et al. 2010; Zhong et al. 2002). Kinesin proteins are involved in many critical cellular processes, including cell elongation, cell-cycle progression, and cell wall biosynthesis (Li et al. 2011; Zhong et al. 2002). In addition to its role as a kinesin, *GDD1* has been shown to bind to the *cis*-element sequence (ACCAACTTGAA) in the *KO2* promoter, which is involved in GA biosynthesis. Mutations in *BC12*/*GDD1* affect GA biosynthesis and gene *KO2* is significantly down-regulated in both mutants *gdd1* and *gs9–1* (Li et al. 2011; Zhang et al. 2010). In contrast, some genes involved in GA biosynthesis, including *KAO*, *GA20ox2* and *GA3ox2,* are up-regulated in mutants *gdd1* and/or *gs9–1*. Based on the observation that the dwarf phenotype of mutant *gs9–1* could be rescued by adding exogenous GA_3_, we hypothesize that the GA levels in mutant *gs9–1* are reduced. In Arabidopsis, *FRA1* is the predicted ortholog of *BC12*/*GDD1/MTD1/GS9–1*. The *fra1* mutant displays phenotypes similar to the *gs9–1* mutant. *FRA1* affects cellulose microfibril orientation and wall composition, resulting a significant reduction in cell number and length of root, shoot, panicle and grain (Zhong et al. 2002).

The phenotypic differences in mutants *bc12*/*gdd1/mtd1/gs9-1* are likely due to the mutated effect of different alleles *BC12*/*GDD1/MTD1/GS9–1.* In the *bc12–1* mutant, the 26-bp deletion at the 4th exon of *BC12* leads to a frameshift mutation and truncates the protein at its 185th amino acid (Fig. 4c, d). The *bc12–1* mutant displays a height less than half of the wild-type plant resulting from a decrease in the longitudinal parenchyma cell number in stem and root (Zhang et al. 2010). A similar frameshift mutation in the *gdd1* mutant causes a 70% reduction in plant height (Li et al. 2011). The *mtd1* mutant, which carries a single nucleotide substitution (G to A) that leads to a nonsense mutation (Fig. 4c, d), displays dwarfism (50% shorter than WT) and increased tillering (Yu et al. 2016). The dwarf traits of *gdd1* and *mtd1* can be rescued by exogenous GA_3_ at concentrations higher than 1 μM (Li et al. 2011; Yu et al. 2016). In contrast to the severe phenotypes reported for the *bc12–1, gdd1* and *mtd1* mutants*,* the *gs9–1* mutant, harboring two amino acid changes in a conserved protein motif (Fig. 4a, e), displays only a 20% decrease in plant height (Fig. 2b, c). The dwarf phenotype of the *gs9–1* mutant can be rescued by adding exogenous GA_3_ at a concentration of 0.1 μM. The phenotypic differences of mutants *bc12*/*gdd1/mtd1/gs9-1* may be due to the diverse natures of the mutations in each allele or differences in the genetic background of each of the mutants or both.

## Conclusions

We identified a grain shape mutant, *gs9–1*, from an FN-induced mutant collection, and identified the gene controlling the *gs9–1* phenotype using whole-genome sequencing. The *gs9–1* gene is an allele of *BC12/GDD1/MTD1*. *gs9–1* is a semi-dominant gene that carries a two-amino acid change, resulting in a 9.0% reduction in cell length and a 7.6% reduction in cell number along the vertical axis of the glume. The reduction of cell number and length in the *gs9–1* mutant is significantly less severe than the changes observed in the *BC12/GDD1/MTD1* mutants. The dwarf trait the *gs9–1* mutant was rescued by adding exogenous GA_3_. In addition to revealing a novel allele of LOC_Os09g02650 controlling grain shape, this study demonstrates the efficiency and convenience of cloning genes from the Kitaake whole-genome sequenced mutant population.

## Materials and Methods

### Plant Materials

Kitaake is an early-flowering *japonica* rice variety (Kim et al. 2013), and X. Kitaake is a line of Kitaake carrying the Xa21 gene under control of the maize ubiquitin promoter (Park et al. 2010). The X. Kitaake seeds were mutagenized by FN irradiation and the mutant population was developed (Li et al. 2016, 2017). A grain shape mutant FN60–4, named *gs9–1*, was discovered in the M_2_ mutant population. Kitaake was used to cross and backcross with *gs9–1*, and their F_1_, BC_1_, BC_1_F_2_, BC_2_F_2_ and BC_2_F_3_ populations were developed.

### Cultivation and Management of Plant Materials

Plants M_2_, M_3_, M_4_, M_5_ and BC_1_F_2_ were planted in greenhouse 715 at the University of California, Davis. The daytime temperature (6 AM to 10 PM) of the greenhouse was set to 80–85 °F, and the night time temperature to 66–68 °F. The humidity range was 40% to 60%, during the day and night. From October 1 to April 30, the supplement of artificial lights (1000w metal halide bulbs) was automatically on when outside light was below 600 W/square meter. Pots (Disposable 5½ inch square pots) were filled with the “Veggie mix” soil up to 1 in. from the top and then soaked with fertilized water. Three plants were grown in each pot.

Populations BC_1_F_2_, BC_2_F_1_, BC_2_F_2_ and BC_2_F_3_ were grown in the experimental field of Xiamen University, China.

### Gene Cloning Based on Whole-Genome Sequencing

Young leaves of 40 mutant lines were collected from the M_3_ population. Twenty lines were used to make one DNA pool. The genomic DNA of two pools was isolated using the cetyltrimethyl ammonium bromide (CTAB) method (Xu et al. 2012) and subjected to whole-genome sequencing on the Illumina HiSeq 2000 platform according to the manufacturer’s instructions at the Joint Genome Institute (JGI) of the US Department of Energy. Mutated loci were detected by means of sequence alignment between the mutant lines and X. Kitaake. X. Kitaake was previously sequenced (Li et al. 2016, 2017). Sequence data used in this study are available from the JGI website (https://genome.jgi.doe.gov) and have been submitted to NCBI’s short read archive under the accession numbers of W60–4H and W60–4 M.

Mutant DNA pools and wild type DNA pools developed from F_2_, BC_1_F_2_, BC_2_F_2_ and BC_3_F_2_ segregation population were used to screen the mutant loci, so as to find target mutant locus which is a cosegregation factor with the target trait.

### Primer Design and Synthesis

Primers used in this study were designed using Primer Premier 6.0 (PREMIER Biosoft, USA) and synthesized at Sangon Biotech (Shanghai, China) Co., Ltd. All primer sequences are listed in Additional file 1: Tables S4 and S5.

### Examinations of Glume Epidermis Cell Size and Number Using the Scanning Electron Microscope (SEM)

Ten rice grains were randomly selected from each rice line and laid flat side by side. The glume epidermis cells in the middle part of the grain were scanned using the Hitachi scanning electron microscope (MT-1000). The size of each view was 15 cm × 20 cm. The magnification was 300 times. The number of glume epidermis cells of every row and column in each view was counted. Length of the glume epidermis cell on the horizontal axis (cell width) was calculated by dividing 20 cm by the number of cell columns. Length of the glume epidermis cell on the vertical axis (cell length) was calculated by dividing 15 cm by the number of cell columns. The average value of glume epidermis cell length was based on 10 rice grains.

### Significance Test

Statistical Product and Service Solutions (SPSS) is a statistical analysis software of the IBM company in USA. In this study, IMB SPSS Statistics 19 was used for the significance test in this study. The significance test of normal distribution was performed based on the ‘Sample K-S’ method, the significance test of multiple sets of data based on the ‘One-Way ANOVA’ method, and the mean difference test of two sets of data based on the ‘Independent-Samples T test’ method.

### GA Induction in Cell Elongation

Seeds of Kitaake and *gs9–1* (*n* = 60) were surfaced sterilized with H_2_O_2_ solution (1%) for 15 min and rinsed with sterile distilled water for three times. The sterilized seeds were then placed on agar plates supplemented with GA_3_ at various concentrations (0, 0.1, 1, 10, 100, 200, and 300 μM) and were grown in a chamber (MGC-250, Shanghai bluepard instruments Co.,ltd., Shanghai, China) at 28 °C and with the day/night period (12/12 h) (12000LX). The length of the 2nd leaf sheath was measured at 8 days after GA3 application.

### qRT-PCR Analysis

Total RNA was isolated from the young panicle at its differentiation stage V using the TaKaRa MiniBEST Plant RNA Extraction Kit (Takara Bio Inc., Japan). The Thermo Scientific RevertAid First Strand cDNA Synthesis Kit was used to synthesize first strand cDNA. SYBR Green II real time PCR was carried out using the TransStart® Top Green qPCR Super Mix Kit (TransgGen Biotech, China) on an ABI Prism 7500 Sequence Detector. The real time PCR amplification mixture (20 μL) contained 1 μg of cDNA, 10 μL of 2× TransStart® Top Green qPCR Super Mix Kit, 0.4 μL of 50× Dye II and 4 μL of 5 μM forward and reverse primers. The relative quantification of each transcript of different genes was calculated using the 2^-ΔΔCT^ method, normalized to the internal control actin gene (LOC_Os03g50885) (Li et al. 2018).

## Additional Files

Additional file 1: **Table S1.** Grain length in parents, mutant lines and populations. **Table S2.** Grain shape and plant height traits of three groups of the BC_3_F_2_ population. **Table S3.** Mutation sites in FN60–4 based on whole genome sequencing. **Table S4.** Primers designed for analyzing mutation sites. **Table S5.** Primers designed for qRT-PCR. **Figure S1.** Distribution of GL (a), GW (b), L/W (c), KGW (d) and PH (e) in the BC_3_F_2_ population. **Figure S2.** Chromosomal distribution of short reads of the FN60–4 W and X. Kitaake with reference to the Nipponbare genome. **Figure S3.** The grain shape of Kit and a *gs9–1* line in BC_2_F_3_. (a) the grain width; (b) the grain length. **Figure S4.** Motif analysis of 9 orthologous genes from 8 species by MEME. (DOC 1890 kb)

Additional file 2: **Table S6.** The phenotype of Kitaake and *gs9–1*. **Table S7.** Results for BLASTP against *Oryza sativa* Japonica Group IRGSP-1.0 (Proteins). (XLSX 25 kb)

## Data Availability

The datasets used and analyzed during the current study are available from the corresponding author on reasonable request.

## Abbreviations

CNVs: Copy number changes
CTAB: Cetyltrimethyl ammonium bromide
EMS: Ethyl methane sulfonate
FN: Fast-neutron
GA: Gibberellic acid
*gs9–1*: *grain shape 9–1*
InDel: Insertion-deletion
KGW: 1000-grain weight
L/W: Grain length-to-width ratio
NGS: Next-generation sequencing
SEM: Scanning electron microscope
SNVs: Single nucleotide variants
WGS: Whole-genome sequencing
WT: Wild type
