# Supplementary material for: Whole-Genome Sequencing Identifies a Rice Grain Shape Mutant, gs9–1
Source: Rice (N Y). 2019 Jul 18;12:52. doi: 10.1186/s12284-019-0308-8 (PMC6639446; doi:10.1186/s12284-019-0308-8)
Supplement: Supplementary file 1 — Table S1. Grain length in parents, mutant lines and populations. Table S2. Grain shape and plant height traits of three groups of the BC3F2 population. Table S3. Mutation sites in FN60–4 based on whole genome sequencing. Table S4. Primers designed for analyzing mutation sites. Table S5. Primers designed for qRT-PCR. Figure S1. Distribution of GL (a), GW (b), L/W (c), KGW (d) and PH (e) in the BC3F2 population. Figure S2. Chromosomal distribution of short reads of the FN60–4 W and X. Kitaake with reference to the Nipponbare genome. Figure S3. The grain shape of Kit and a gs9–1 line in BC2F3. (a) the grain width; (b) the grain length. Figure S4. Motif analysis of 9 orthologous genes from 8 species by MEME. (DOC 1890 kb) [file 12284_2019_308_MOESM1_ESM.doc]

Additional file 1

**Table S**1 Grain length in parents, mutant lines and populations

| Line & popu. | Mean  (mm) | SD | Min  (mm) | Max  (mm) | Number |
| --- | --- | --- | --- | --- | --- |
| X.Kit | 7.04 | 0.18 | 6.80 | 7.28 | 6 |
| Kit | 7.08 | 0.03 | 7.03 | 7.13 | 5 |
| *gs9-1* | 6.12 | 0.22 | 5.55 | 6.45 | 28 |
| F_1_ | 6.65 | 0.10 | 6.43 | 6.78 | 12 |
| BC_1_F_1_ | 6.73 | 0.24 | 6.05 | 7.10 | 17 |

Note：F_1_(from the cross between FN60-4 and Kit) and BC_1_F_1_(from the cross between F_1_ and Kit).

**Table S2 Grain shape and plant height traits of three groups of the BC_3_F_2_** population

| Group^*^ | GL | GW | L/W | KGW | PH | Plant^#^ Number | Group Standard  （mm) |
| --- | --- | --- | --- | --- | --- | --- | --- |
|  | (mm) | (mm) |  | (g) | (cm) |  |  |
| WT | 7.06±0.02A | 3.31±0.01C | 2.14±0.01A | 28.0±0.2A | 66.1±0.6A | 17 | L/W≥2.11 |
| H | 6.74±0.01B | 3.43±0.01B | 1.97±0.01B | 27.2±0.1B | 62.7±0.6B | 35 | 1.91≤L/W≤2.04 |
| M | 6.12±0.02C | 3.57±0.02A | 1.72±0.01C | 24.3±0.2C | 53.1±0.8C | 12 | L/W≤1.75 |

Not:^*^WT, H and M mean wild-type, heterozygous an *gs9-1* genotypes, respectively. GL, Grain Length; GW, Grain Width; L/W, The ratio between GL and GW; KGW, The weight of 1000 grains, PH, Plant Height. The capitals after the mean value show the significance level of anova between three groups, and the same capitals between two groups means no significant difference and the different capitals between two groups means highly significant difference. ^#^ χ_c_^2^=1.21<χ_0.05,2_^2^, χ_0.05,2_^2^=5.99. This result shows the grain shape (L/W) of this population fits the semi-dominant gene segregation ratio of 1:2:1.

**Table S**3 Mutation sites in FN60-4 based on whole genome sequencing

| Chrom. |  | Pos. | WT | Mutant | Deletion（bp) | Mutant type | Genotype | Genes affected |
| --- | --- | --- | --- | --- | --- | --- | --- | --- |
| Chr1 |  | 14890045 | G | A | \ | SNP | 1/1 | \ |
| Chr1 |  | 16738881 | C | A | \ | SNP | 1/1 | \ |
| Chr1 |  | 25369840 | C | T | \ | SNP | 1/1 | LOC_Os01g44250 |
| Chr1 |  | 43025746 | A | G | \ | SNP | 0/1 | \ |
| Chr2 |  | 2460820 | AGAG | AGA | \ | INDEL | 1/1 | -208 bp of LOC_Os02g05150 |
| Chr2 |  | 4517203 | A | T | \ | SNP | 1/1 | \ |
| Chr2 |  | 22258901 | (TC)16 | (TC)13 | \ | INDEL | 0/1 | LOC_Os02g36880 |
| Chr3 |  | 487223 | G | A | \ | SNP | 1/1 | \ |
| Chr3 |  | 6339372 | G | T | \ | SNP | 0/1 | LOC_Os03g12080 |
| Chr3 |  | 10769497 | G | A | \ | SNP | 0/1 | \ |
| Chr3 |  | 16728966 | C | T | \ | SNP | 0/1 | \ |
| Chr3 |  | 18649273 | AAGGAGGA | AAGGA | \ | INDEL | 0/1 | \ |
| Chr3 |  | 24301250 | (T)8 | (T)7 | \ | INDEL | 1/. | \ |
| Chr3 |  | 26216738 | G | T | \ | SNP | 0/1 | -196 bp of LOC_Os03g46350 |
| Chr3 |  | 27094253 | ATTTCCGG | A | \ | INDEL | 0/1 | LOC_Os03g47754 |
| Chr3 |  | 34742202 | T | A | \ | SNP | 1/1 | \ |
| Chr4 |  | 4520977 | G | C | \ | SNP | 0/1 | LOC_Os04g08415 |
| Chr4 |  | 12882299 | A | G | \ | SNP | 1/1 | LOC_Os04g22720 |
| Chr4 |  | 32012100 | (A)9 | (A)10 | \ | INDEL | 1/1 | LOC_Os04g53720 |
| Chr12 |  | 9469128 | ACGCT | ACGC | \ | INDEL | 1/. | -810 bp of LOC_Os12g16524 |
| Chr12 |  | 18170447 | ATCTGAAAC | AT | \ | INDEL | 1/1 | \ |
| Chr12 |  | 26338896 | (A)12 | (A)11 | \ | INDEL | 1/. | \ |
| Chr9 |  | 1164547 | TTAATC | TTA | \ | INDEL | 1/1 | LOC_Os09g02650 |
| Chr5 |  | 18028739 | \ | \ | 10 | DEL | 1/. | \ |
| Chr5 |  | 24996046 | \ | \ | 20 | DEL | 1/. | \ |
| Chr9 |  | 282372 | \ | \ | 71052 | DEL | 1/1 | LOC_Os09g01360 LOC_Os09g01370  LOC_Os09g01375  LOC_Os09g01380  LOC_Os09g01390 LOC_Os09g01400 LOC_Os09g01410  LOC_Os09g01420 LOC_Os09g01430 LOC_Os09g01440 LOC_Os09g01450  LOC_Os09g01460 LOC_Os09g01470 LOC_Os09g01480 |
| Chr9 |  | 2682848 | \ | \ | 25 | DEL | 1/1 |  |
| Chr9 |  | 6716773 | \ | \ | 31 | DEL | 1/1 |  |
| Chr9 |  | 20288296 | \ | \ | 9516 | DEL | 1/1 |  |
| Chr11 |  | 26139294 | \ | \ | 57 | DEL | 1/1 |  |

**Table S4 Primers designed for analyzing** mutation sites

| Genes | Name of primer | Sequence | PCP product size (bp) |
| --- | --- | --- | --- |
| LOC_Os01g44250 | W60-44250F | GCGTGCTGCCATTAGTTCAG | 1086 |
|  | W60-44250R | CAGGCTTGGTCGTATTTGCG |  |
| LOC_Os02g05150 | W60-05150F | GAATGTGTTTCGTCGGGTCTC | 625 |
|  | W60-05150R | TTGCGTGCTTCCTCATCAAG |  |
| LOC_Os04g22720 | W60-22720F | TGGAGACACAGCCACTATACACA | 619 |
|  | W60-22720R | GCCAGAGGTCATCATTCTCACAA |  |
| LOC_Os04g53720 | W60-53720F | TGGAATTTGTGAAGGTGGGAAAC | 913 |
|  | W60-53720R | GTCGCAGCAGCAATACAAGAATG |  |
| LOC_Os09g02650 | W60-02650-2F | TGCCAACCTTGTTCCATTATCAG | 907 |
|  | W60-02650-2R | ACTTACATGAGAGCCAGGAGAAG |  |
| LOC_Os09g01360 | W60-01360F | TGGTCCTCCTTGGCAGTATCAG | 974 |
|  | W60-01360R | GTGGTAGTGATTGGTGGTTGAAGT |  |
| LOC_Os09g01370 | W60-01370F | CCAAGCCAACCAAGGTGAATC | 1970 |
|  | W60-01370R | GTAGCAATCAGGCAGGACAC |  |
| LOC_Os09g01375 & LOC_Os09g01380 | W60-01375F | TCCGAATCCACACCTACAGTCC | 2209 |
|  | W60-01375R | CTTGCTAGAGTGCGATCTGAGG |  |
| LOC_Os09g01390 | W60-01390F | CGTGCGAGGATTTGATGATAGC | 1252 |
|  | W60-01390R | GCGAGAATACAGGCTCTGGAAG |  |
| LOC_Os09g01400 | W60-01400F | GTGGATTTGGTGTTTGAGGGC | 2095 |
|  | W60-01400R | GTCAGATGGTGGTTTCTCCGA |  |
| LOC_Os09g01410 | W60-01410F | AGAGAGCGGCGGGATTCAAA | 1978 |
|  | W60-01410R | AAGAAGGCGGTGGAGATAGAGG |  |
| LOC_Os09g01420 | W60-01420F | TCAACCGCCTCTATCTCCAC | 2199 |
|  | W60-01420R | ACAAGCGAATGACTGCCGA |  |
| LOC_Os09g01430 | W60-01430F | CTACATCAACCCAAGCCGAC | 2388 |
|  | W60-01430R | CGAGAAGAAGCACGAGTGGT |  |
| LOC_Os09g01440 | W60-01440F | CTTGGTGGTTGTGGATTTGGT | 1917 |
|  | W60-01440R | TGAGATGGTGGTTTGAGCCTG |  |
| LOC_Os09g01450 | W60-01450F | CAGAACAGCCGAAGAAGGAGT | 2044 |
|  | W60-01450R | CACGATGCCCAAGTTTAGGTG |  |
| LOC_Os09g01460 | W60-01460F | TAGTGAGGGTGGAGTTGCGT | 1388 |
|  | W60-01460R | AGCCGAAGAAGGAGTCTGGA |  |
| LOC_Os09g01470 | W60-01470F | GCGGTGTACTGACGGATTCTAT | 2115 |
|  | W60-01470R | ATGTAGACTCTGCTCTGCTTCA |  |
| LOC_Os09g01480 | W60-01480F | CTCGTTGAGCATCGGCTTCCA | 1579 |
|  | W60-01480R | GCACCACCAATTCCGAATCACC |  |

**Table S5 Primers designed for qRT-PCR**

| Genes | Name of primer | Sequence | reference |
| --- | --- | --- | --- |
| *gs9-1* | q02650F | CAAGCACTTAATCAGCGGCAGAA | \ |
|  | q02650R | GAGAGCCAGGAGAAGTACCATTCA |  |
| *Actin* | qActin-F | CTGCTATGTACGTCGCCATCCAG |  |
|  | qActin-R | TGAGATCACGCCCAGCAAGGT |  |
| *CESA6* | qCESA6-F | TCCCTGGACATCCATTCCACTTCT |  |
|  | qCESA6-R | TGCTCATTCCTCCACCAGTCATC |  |
| *KAO* | qKAO-F | CGCAAGAGCAAAGGCTGAG | [Li et al. 2011](#_ENREF_18) |
|  | qKAO-R | CCTGTGAGAGGAAGTGCATCTTCT |  |
| *KO2* | qKO2-F | TGAAGTAGCCAAGGAGGCGA |  |
|  | qKO2-R | CGCTGATTGCGACCATACTTT |  |
| *GA3ox2* | qGA3ox2-F | TCTTCTCCAAGCTCATGTGGT |  |
|  | qGA3ox2-R | AACTCCTCCATCACGTCACAG |  |
| *GA20ox2* | qGA20ox2-F | GGGAGGGTGTACCAGAAGTACTG |  |
|  | qGA20ox2-R | GGCTCAGCTCCAGGAGTTCC |  |
| LOC_Os05g03190 | 03190F | CGTCATTGCCACCTGGTGTATCA | \ |
|  | 03190R | CCTCCTTGAACGTATCTGCGACTG |  |
| LOC_Os12g05900 | 05900F | CACGTCACCTTCGATAGGTTCCAT |  |
|  | 05900R | CCGAAGACCGCCTTGTTCATAAAG |  |
| LOC_Os03g13460 | 13460F | ATGCGTGTGTACCGTCGAAAGG |  |
|  | 13460R | AGATCTTCCAGTAGAGGCGTCACT |  |
| LOC_Os06g49320 | 49320F | TCACCTACATCAAGGCTCGCTACT |  |
|  | 49320R | TCTTGGAGTGGTTGCCGTCGA |  |
| LOC_Os03g56260 | 56260F | GCAAGCATTAACATCACGGAGACA |  |
|  | 56260R | GCTCGGCATTAGTCATCCCAAGAA |  |
| LOC_Os03g60340 | 60340F | GCCATAATGCAGCGGGCTCT |  |
|  | 60340R | AGGTGAGGTCGGGCTGGATGTA |  |
| LOC_Os01g64490 | 64490F | AATGGATTCAAGGAGGAGGCAAGC |  |
|  | 64490R | GCCAACCAGATTGCTGTTTCTCG |  |
| LOC_Os01g67190 | 67190F | GGGCAATGCCATCTATTGTGTGT |  |
|  | 67190R | CTGTAGAATTGCCAAATGCGTGTG |  |
| LOC_Os10g21940 | 21940F | CGCGACAGCACGGGAATTACTT |  |
|  | 21940R | ACTGTGCGATACAGTCAACAACAG |  |

Li J, Jiang J, Qian Q, Xu Y, Zhang C, Xiao J, Du C, Luo W, Zou G, Chen M, Huang Y, Feng Y, Cheng Z, Yuan M, Chong K (2011) Mutation of Rice BC12/GDD1, Which Encodes a Kinesin-Like Protein That Binds to a GA Biosynthesis Gene Promoter, Leads to Dwarfism with Impaired Cell Elongation. The Plant Cell 23:628-640

**Additional Figure**

**
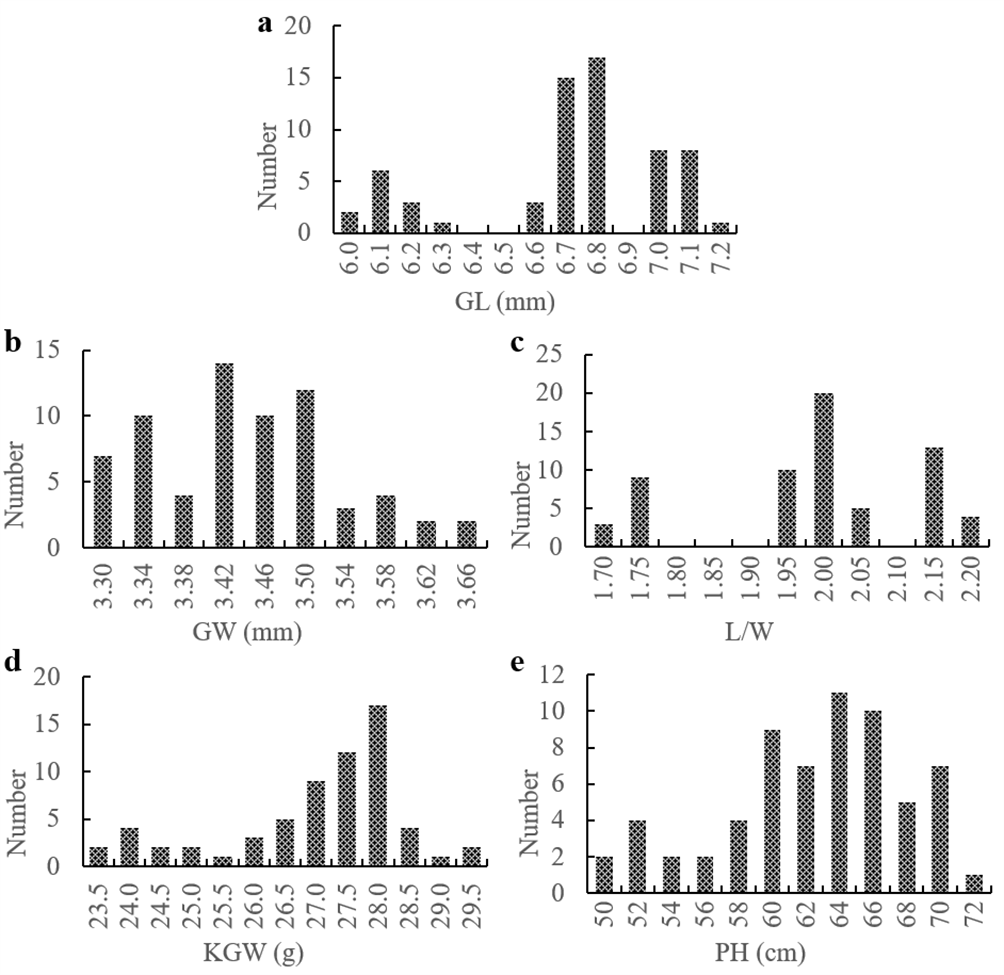
**

**Figure S1 Distribution of GL (a), GW (b), L/W (c), KGW (d) and PH (e) in the BC_3_F_2_ population.**

**
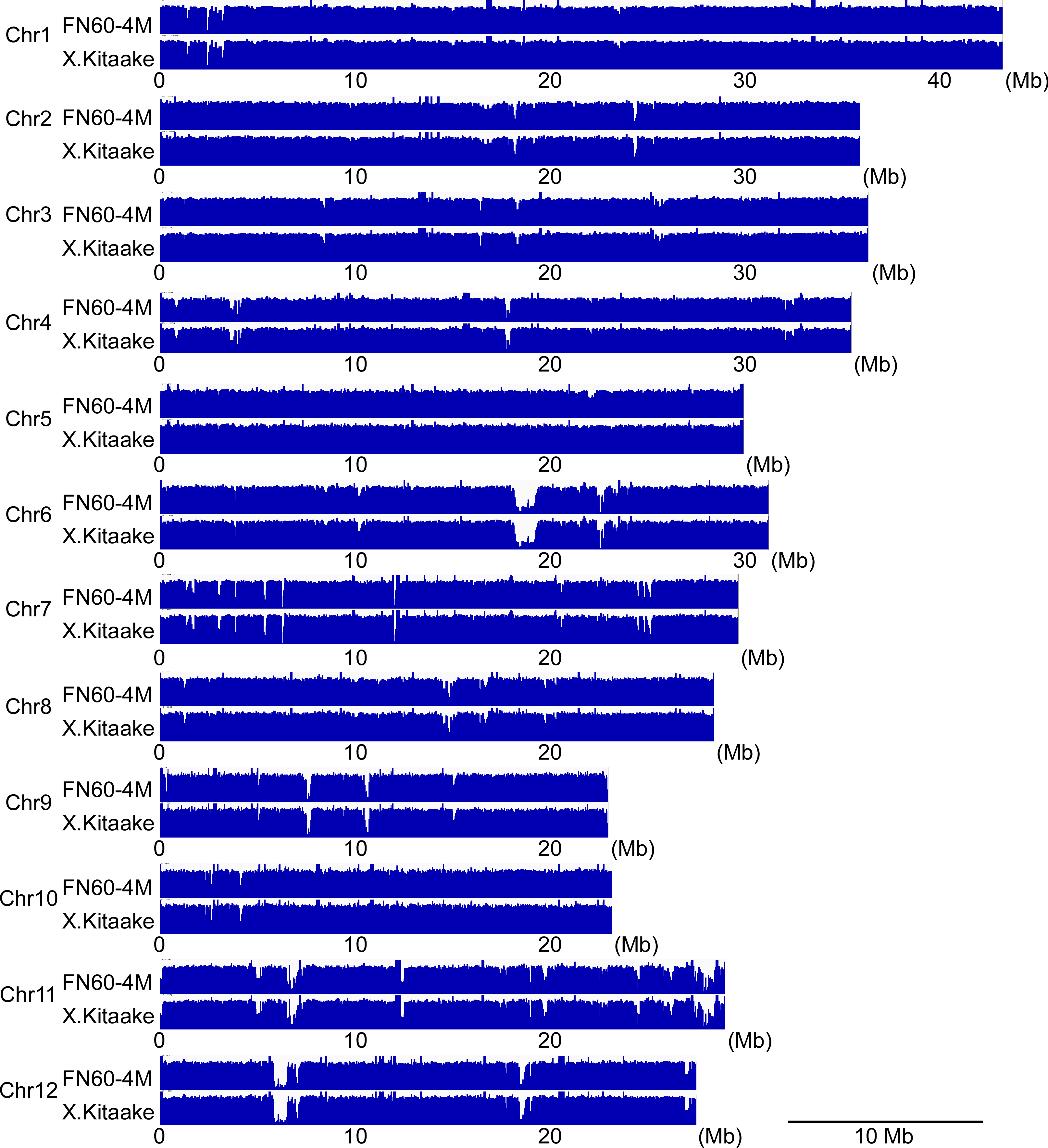
**

**Figure S2 Chromosomal distribution of short reads of the FN60-4W and X.Kitaake with reference to the Nipponbare genome.** The diagram was generated using Integrative Genomics Viewer (IGV) Tools from BAM files with bin size of 1000 bp and visualized using IGV. The maximal cutoffs of sequencing depth for FN60-4W and X.Kitaake are 50x and 100x, respectively.


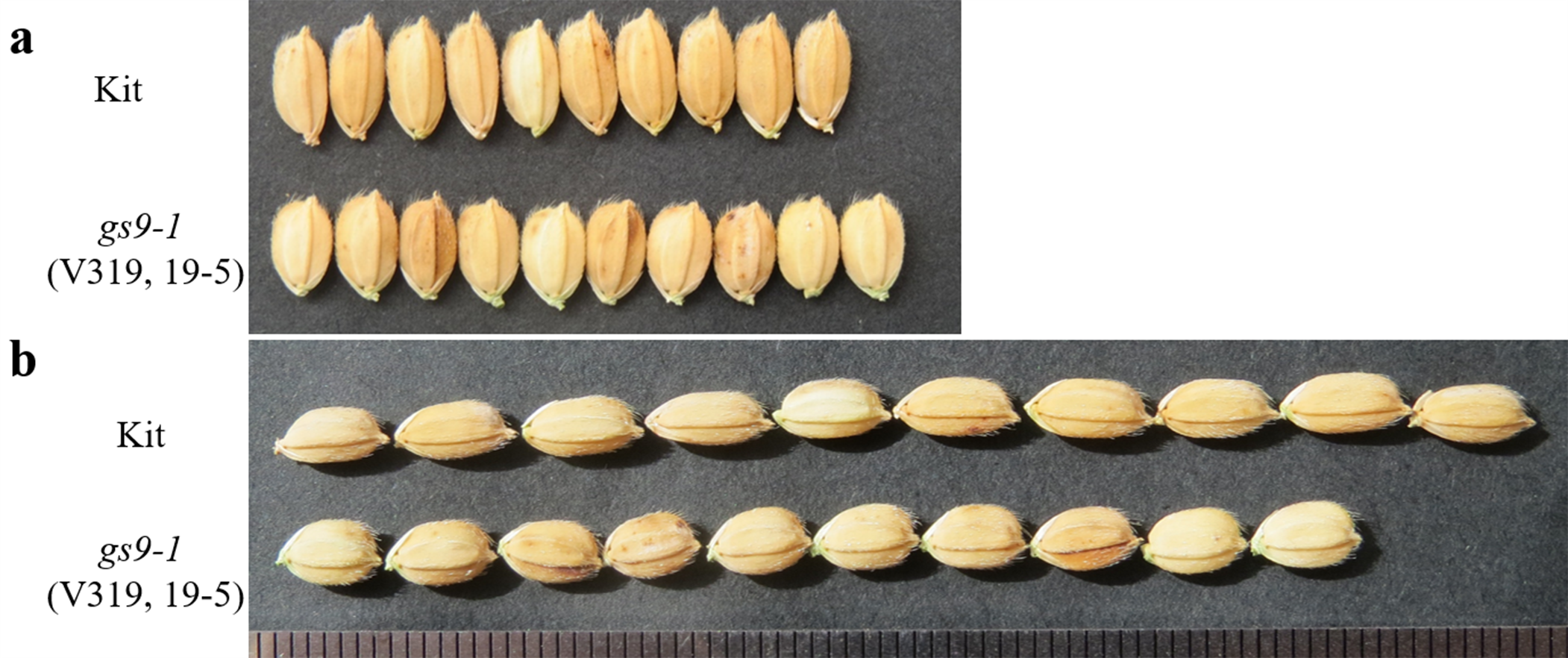


**Figure S3 The grain shape of Kit and a *gs9-1* line in BC_2_F_3_. (a) the grain width; (b) the grain length.**


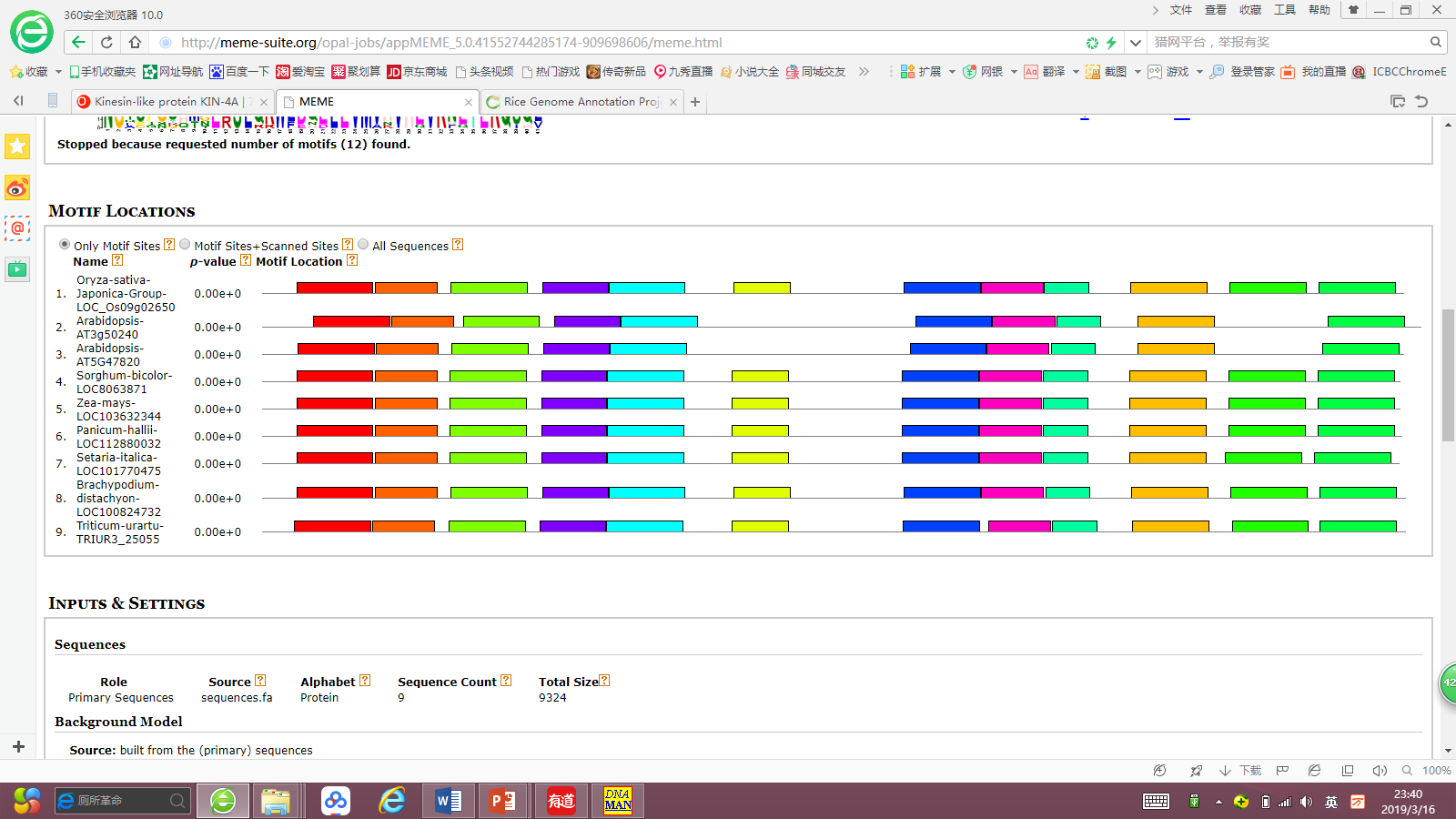


**Figure S4 Motif analysis of 9 orthologous genes from 8 species by MEME** (Multiple Em for Motif Elicitation, <http://meme-suite.org/tools/meme>). Every rectangle shows one motif and different color means different motifs. The black triangle points to the mutation site.

**Query condition**

Query sequence: the 9 protein sequences of LOC_Os09g02650 orthologous genes from 8 species.

Select the number of motifs:12

How wide can motifs be? Minimum width: 6 and Maximum width:70.

Other condition: default.
